# On a Peculiar Form of Malignant Inflammation of the Lips and Face, Resembling Malignant Pustule

**Published:** 1854-07

**Authors:** W. Parker

**Affiliations:** Professor of Surgery in the College of Physicians and Surgeons, New York.


### ARTICLE XV.

*On a Peculiar Form of Malignant Inflammation of the Lips and Face, resembling Malignant Pustule.* By W. PARKER, M. D., Professor of Surgery in the College of Physicians and Surgeons, New York.

THERE have come under my observation, within a recent period, several cases of a peculiar form of inflammation of the lips and face, which resembles somewhat phlegmonous erysipelas, but more strikingly, especially in its commencement, malignant pustule, and, in its subsequent progress, carbuncle. It, however, differs from these affections in some essential particulars, which will be noticed after giving the details of the following cases, which illustrate the peculiarity of this form of disease.

Case 1.—I first saw this patient on the 18th of last December. He was a young man, aged 23, merchant, of good character, temperate habits, and in the previous enjoyment of good health. About a week before I visited him, a small pustule made its appearance upon the central portion of the lower lip, just below the edge of the vermilion border. It became painful, had a livid areola, gradually but slowly enlarged, and finally broke and began to discharge. The pain increased, and the swelling extended downwards upon the chin. At my first visit, about this period, the tumefaction had reached as low as the os hyoides, and had extended over the right side of the face to the head; it was hard to the feel, of a livid color, insensible, and had now much the appearance of a carbuncle. The lips were greatly tumefied, everted; gums swollen, and of the same livid color; tongue moist; inside of mouth unaffected; ptyalism considerable. The lower lip, about the seat of the original pustule, appeared gangrenous. The pulse was 120, rapid and feeble, respiration unaffected. He was able to get up and sit in the chair, but was suffering from great depression of the vital powers. The course pursued consisted of deep scarifications of the lips and yeast poultices to the swelling, and stimulants to sustain the general system. The swelling continued to extend, involving successively the neck, face, and finally the head. He died on the following day, the 19th, late in the evening.

Case 2.—I visited, on the 15th of January, a patient, aged 45, merchant, suffering from what appeared to be a carbuncle of the under lip. He was of a good constitution, temperate habits, and in the enjoyment of good health, previously to the present attack. Four days before I saw him, he was supposed to have cut the lower lip slightly, and applied to it arnica. The inflammation commenced at this point, the lip swelled largely, became everted, had a livid color, was tender, hard, and the seat of a burning pain. At several points there were small sloughy apertures, discharging thin pus. The constitutional symptoms were considerable, but not sufficient to confine him to his room. The treatment consisted of free incision and yeast poultices to the lip, and sustaining remedies for the general system. Portions of the lip sloughed, but he recovered.

Case 3.—Mr. W., aged 26, married, furniture dealer, of good habits, and hitherto perfect health, discovered a small pustule on the under lip near the right angle of the mouth, on the 2d of April. It was tender on pressure and had a hard base, but attracted no other attention. During the night the disease extended considerably, involving the whole lip and the right side of the face in a hard, livid, and painful swelling. On the evening of the second day his physician first saw him, and found the lip greatly swollen, of a livid color, and the seat of a burning pain. He scarified the parts for the purpose of local depletion, and also applied leeches. The swelling continued to extend, involving the right side of the neck and face to a great extent. I saw him on the 7th, at 11 A. M. His symptoms were then most unfavorable, pulse 130 per minute, intermittent every seventh or eighth beat, weak and small; respiration rapid, moaning; skin warm and moist; urine free; pupils much dilated; mind clear. He complained of oppression about the chest, and had not been able to obtain sleep. Both lips were involved in the swelling; were hard, livid, and insensible; the whole side of the neck and face was similarly affected, the eye being nearly closed. The frontal vein was livid, red, and prominent, and the veins of the cheek were also visible, as if distended. The treatment consisted of deep scarifications of the lips, and yeast poultices to the part, with anodynes and stimulants. I visited him again at six o'clock, P. M., and found him rapidly failing; treatment of no service. He died the same evening.

Case 4.—I was called, April 10th, to see Miss S., aged 30, occupied as a governess, of good constitution, whom I found laboring under the same difficulty as in the preceding cases. Her history was almost precisely similar. Five days before, while in the possession of apparently perfect health, she first observed a small pustule on the lower lip, just below the red line of mucous membrane; it was regarded as a small boil, and no attention given to it. On the following day the pustule had enlarged somewhat, was hard, and had a livid areola, but she continued about her employment; she spent a feverish, restless night, and on the next day called her physician. The disease gradually extended, assuming the appearances already noticed, and for two days no danger was apprehended. Her symptoms now became much more unfavorable, and at this period I first saw her. She was lying in bed quite insensible; deglutition difficult; respiration laborious; right side of the body paralyzed; lips large, everted, and cold; right side of face, neck, and forehead swollen like the lip, hard and purple; right eye protruded; pupils dilated and insensible. On making an incision into the lip, the cellular substance was found filled with small deposits of pus, which were forced out on slight pressure. As she was moribund, treatment was of no avail.

From the history of the foregoing cases, it is evident that this disease differs from erysipelas, for which it has in several instances been mistaken, in its origin in a pustule, without a chill or other constitutional disturbance, the hardness of the swelling, its purple or livid color, insensibility, and absence of much pain. It differs from carbuncle, which in some features it resembles in the class of individuals which it attacks—they being young, temperate, of sound constitution, and in the previous enjoyment of good health—and in its rapidly fatal course. Carbuncle, on the contrary, occurs by preference in persons enfeebled by age or vicious habits. It differs again from true malignant pustule, to which, in its origin, it seems allied, by attacking persons who have not been affected by poisonous wounds, or who have been liable to the introduction of animal poisons into the system.

This disease would therefore seem to be peculiar, having many points of resemblance to other similar affections, but still not so closely allied to any one as to warrant its classification under the same head. In every instance which has come under my own observation, the pustule has been seated upon the lower lip, and from this point the inflammation has spread. In a fatal case related to me by a physician, in whose practice it recently occurred, the pustule was seated upon the side of the nose.

Although the nature and progress of the disease show a vitiated state of the system, in no instance have I been able to trace the attack to the contact of poisonous matter, or its reception into the system in the food or drink. In every instance the patient has been in the enjoyment of good health, and the progress of the disease, though rapid, has excited so little local and general disturbance as not to excite alarm until a short time before its fatal termination. The general symptoms are of a typhoid character, the vital powers being evidently depressed either by the influence of the disease itself, or, which is more probable, the cause upon which the development of the disease depends.

The late Dr. Peirson, of Salem, Mass., reported (*Bost. Med. and Surg. Jour.*, 1852,) several cases very similar to the above, and considers the disease malignant pustule. Among them is the case of Hon. Robert Rantoul, whose disease was thought to be erysipelas, but which Dr. Peirson describes as malignant pustule. The pustule in this instance was situated upon the forehead, and depended upon no known local cause. With but one or two exceptions, the remaining cases in this paper occurred in curriers, and hence Dr. Peirson attributes the disease to inoculation with dead animal matter. Some of them bear a strong resemblance to the cases above related, the disease attacking the lips of healthy young persons, entirely unexposed, and spreading thence upon the face. These can scarcely be classified under the head of malignant pustule as described by authors. Bayle speaks of a form not depending upon external cause, but this distinction is not generally received.

The success of the treatment depends upon the *early* recognition of the true nature of the disease. It is very liable to be mistaken for erysipelas, and a course of treatment adopted accordingly, which avails little in staying its progress. Attention to the points of a differential diagnosis already given, will prevent the practitioner from falling into this error. The treatment best adapted to meet the indications of the case, are deep and free scarifications, followed by yeast poultices, or turpentine, the object being to prevent sloughing and to promote healthy suppuration. The general system requires soothing and sustaining remedies, such as are suited to an ataxic condition. The early and prompt employment of these means will afford a fair, and probably the only, hope of success in the treatment of this disease.

My object in this communication is to call special attention to this disease, which, from the frequency of its occurrence in my own practice, and other cases which have been related to me, seems to be very prevalent. The points of particular interest are in regard to its causes and pathology. Does it depend upon an endemic or epidemic cause, or upon the introduction of animal poison into the system? Is there phlebitis as a cause or consequence? These questions are important, and can only be settled by careful observation made upon the living and dead subject.—*N. Y. Jour. of Medicine.*

